# Integrated Proteomics and Transcriptomics Analyses Reveal the Transcriptional Slippage of a Bymovirus P3N-PIPO Gene Expressed from a PVX Vector in *Nicotiana benthamiana*

**DOI:** 10.3390/v13071247

**Published:** 2021-06-26

**Authors:** Chulang Yu, Runpu Miao, Zhuangxin Ye, Stuart MacFarlane, Yuwen Lu, Junmin Li, Jian Yang, Fei Yan, Liangying Dai, Jianping Chen

**Affiliations:** 1 Hunan Provincial Key Laboratory for Biology and Control of Plant Diseases and Insect Pests, College of Plant Protection, Hunan Agricultural University, Changsha 410128, China; yuchulang@nbu.edu.cn; 2 State Key Laboratory for Managing Biotic and Chemical Threats to the Quality and Safety of Agro-Products, Key Laboratory of Biotechnology in Plant Protection of Ministry of Agriculture and Zhejiang Province, Institute of Plant Virology, Ningbo University, Ningbo 315211, China; runpumiao@163.com (R.M.); yzx244522794@163.com (Z.Y.); luyuwen@nbu.edu.cn (Y.L.); lijunmin@nbu.edu.cn (J.L.); nather2008@163.com (J.Y.); yanfei@nbu.edu.cn (F.Y.); 3 Cell and Molecular Sciences, James Hutton Institute, Invergowrie, Dundee DD2 5DA, UK; Stuart.MacFarlane@hutton.ac.uk

**Keywords:** P3N-PIPO, transcriptional slippage, PVX, *Bymovirus*

## Abstract

P3N-PIPO (P3 N-terminal fused with Pretty Interesting Potyviridae ORF), the movement protein of potyviruses, is expressed as a translational fusion with the N-terminus of P3 in potyviruses. As reported in previous studies, P3N-PIPO is expressed via transcriptional slippage at a conserved G_2_A_6_ slippery site in the genus *Potyvirus*. However, it is still unknown whether a similar expression mechanism of P3N-PIPO is used in the other genera of the family *Potyviridae*. Moreover, due to the extremely low expression level of P3N-PIPO in natural virus-infected plants, the peptides spanning the slippery site which provide direct evidence of the slippage at the protein level, have not been identified yet. In this study, a potato virus X (PVX)-based expression vector was utilized to investigate the expression mechanism of P3N-PIPO. A high expression level of the P3N-PIPO(WT) of turnip mosaic virus (TuMV, genus *Potyvirus*) was observed based on the PVX expression vector. For the first time, we successfully identified the peptides of P3N-PIPO spanning the slippery site by mass spectrometry. Likewise, the P3N-PIPO(WT) of wheat yellow mosaic virus (WYMV, genus *Bymovirus*) was also successfully expressed using the PVX expression vector. Integrated proteome and transcriptome analyses revealed that WYMV P3N-PIPO was expressed at the conserved G_2_A_6_ site through transcriptional slippage. Moreover, as revealed by mutagenesis analysis, Hexa-adenosine of the G_2_A_6_ site was important for the frameshift expression of P3N-PIPO in WYMV. According to our results, the PVX-based expression vector might be used as an excellent tool to study the expression mechanism of P3N-PIPO in *Potyviridae*. To the best of our knowledge, this is the first experimental evidence dissecting the expression mechanism of a bymovirus P3N-PIPO in the experimental host *Nicotiana benthamiana*.

## 1. Introduction

Most RNA viruses have a relatively small genome size, which seems to be insufficient for RNA viruses to cope with long-term selective pressure. RNA virus has explored various strategies to maximize its coding capacities, such as splicing, transcriptional slippage, ribosomal frameshifting, leaky scanning, and stop-codon read-through [1,2].

Amongst them, transcriptional slippage often takes place on repetitive nucleotides such as poly(A) or poly(T) tracts, resulting in the synthesis of heterogeneous mRNAs that always insert one or more extra nucleotides but seldom delete one or two bases [3]. If this occurs in a coding sequence, then the heterogeneous mRNA will translate into more than one protein product. It is well-recognized that transcriptional slippage is utilized in bacteria such as *Escherichia. Coli* [3,4]. For example, the expressions of *E. coli* operons, *pyrBI* and *codBA*, were regulated by transcriptional slippage under special conditions [5,6]. As for animal viruses, transcriptional slippage has also been observed in the Ebola virus [7,8,9] (a negative-strand RNA virus) and the Hepatitis C virus (HCV) [10] (a positive-strand RNA virus). With regard to plant viruses, replication slippage(transcriptional slippage)is considered a common evolution process in the genus *Potyvirus* [11], but direct evidence is still missing due to the extremely low expression level of the transcriptional slippage during infection [12].

A short open reading frame (ORF), known as PIPO (Pretty Interesting Potyviridae ORF), is discovered within the genome sequences of *Potyviridae* by bioinformatics analysis [13]. PIPO was proven to be expressed as a part of a fusion protein with the *P3* N-terminal region, named P3N-PIPO [13,14]. Thereafter, numerous studies have been conducted, and the results show that P3N-PIPO is the movement protein of potyviruses [14,15,16,17,18]. High-throughput sequencing has shown that transcriptional slippage at the G_2_A_6_ site accounts for the expression mechanism of P3N-PIPO in potyviruses [2,19]. It is worth noting that previous studies on P3N-PIPO mainly focused on the genus *Potyvirus*, but there are no experimental data supporting whether this mechanism is widely used by the other members in the family *Potyviridae*. Moreover, previous studies suggested that the slippage efficiency varied between 0.8 and 2.1% in potyviruses [2,19]. Owing to the extremely low expression level of P3N-PIPO in natural virus-infected plants, a reliable experimental system is needed to isolate sufficient frameshift-derived protein for analysis. The potato virus X (PVX)-based expression vectors are commonly used exogenous protein expression systems due to their systemic expression and high expression levels in plants [20,21]. 

In this study, the PVX-based vector system was utilized to investigate the expression mechanism of P3N-PIPO. The frameshift expression of P3N-PIPO(WT)-GFP was successfully identified using the PVX-based vector in plants. In addition, the frameshift of P3N-PIPO(WT) in another plant virus, wheat yellow mosaic virus (WYMV) of the genus *Bymovirus*, was also successfully identified and characterized. 

Our research not only sheds more light on the expression mechanism of P3N-PIPO in the family *Potyviridae*, but also provides an excellent tool to study the slippage efficiency of P3N-PIPO.

## 2. Materials and Methods

### 2.1. Construction of PVX-Derived Vectors

The PVX vector (pgR106) used in this study was obtained from David Baulcombe’s laboratory. A *gfp* fragment without the start codon was amplified from a GFP binary vector using specific oligonucleotide primers (Table S1) that incorporated *Asc*I/*SnaB*I and *Sal*I restrictions sites at the 5′- and 3′-terminal, respectively. Then, this *gfp* fragment was cloned into the pgR106 vector in line with the standard molecular clone protocols and was named PVX-GFP (*Asc*I/*SnaB*I, *Sal*I).

Additionally, P3N-PIPO(WT) was amplified from TuMV and WYMV infectious clones, respectively, by the use of specific oligonucleotide primers (Table S1) that incorporated *Cla*I and *SnaB*I restrictions sites at the 5′- and 3′-terminus, respectively. Afterwards, this fragment was cloned into the pJET1.2/blunt vector (Thermo Fisher Scientific, Waltham, MA, USA) following the standard molecular clone protocols and was named the pJET-P3N-PIPO (*Cla*I/*SnaB*I) vector.

The in-frame control construct WYMV P3N-PIPO(FS-1)-GFP was generated by inserting adenosine into the G_2_A_6_ site of WYMV P3N-PIPO(WT) with the QuikChange Lightning Site-Directed Mutagenesis Kit (Agilent Technologies Inc., Palo Alto, CA, USA), according to the manufacturer’s instructions (Table S1). Other mutants like PVX-WYMV-P3N-PIPO-M1 and PVX-WYMV-P3N-PIPO-M2 were constructed by the same method (Table S1).

**Figure 1:**
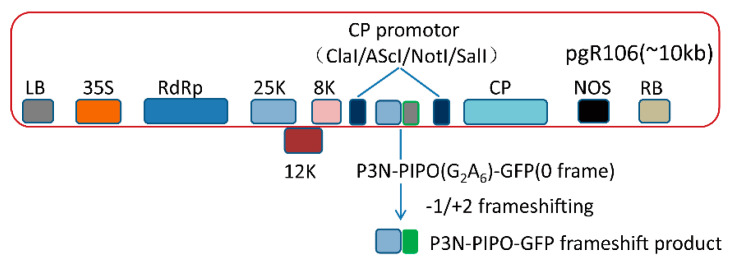
The schematic diagram of P3N-PIPO frameshift expression vector.

First of all, PVX-GFP (*Asc*I/*SnaB*I, *Sal*I) and pJET-P3N-PIPO (*Cla*I/*SnaB*I) were digested with *Cla*I and *SnaB*I, respectively. Then, PVX-GFP (*Cla*I/*SnaB*I) and P3N-PIPO (*Cla*I/*SnaB*I) were purified by gel extraction (Thermo Fisher Scientific, Waltham, MA, USA) and ligated by T4 DNA ligase (Figure 1). 

### 2.2. Growth of Nicotiana Benthamiana Plants, Agrobacterium Infection of Plants, and Confocal Imaging Analysis

*N. benthamiana* were grown in a glasshouse under the following conditions: temperature (day: night) 25 °C: 22 °C ± 2 °C, and 16 h of light. 

All the PVX chimeric virus vectors constructed in this study were transformed into the *Agrobacterium tumefaciens* strain GV3101 (carrying the helper plasmid pSoup), as described previously [22]. Moreover, agroinfiltration of *N. benthamiana* with PVX chimeric virus vectors was also implemented, as described previously [23]. 

Plant leaves expressing recombinant proteins were imaged using a Leica TCS SP2 confocal microscope. Green fluorescent protein (GFP) was excited at 488 nm, and the emitted light was captured at 507 nm. Images were captured digitally and processed using the Leica LCS software.

### 2.3. Western Blotting (WB) Analysis

To analyze the frameshift expression of P3N-PIPO, total proteins were extracted from *N. benthamiana* leaves infected with PVX chimeric viruses using a lysis buffer (100 mM Tris-HCl (pH = 6.8), 20% SDS, and 2% β-mercaptoethanol) and were separated by 12% SDS-PAGE. Proteins were electroblotted onto a preactivated PVDF membrane. Then, the membrane was blocked with block buffer (50 mL 1 × TBS buffer containing 5% skimmed milk powder) and probed with the GFP monoclonal antibody (Sigma-Aldrich Corp., St. Louis, MO, USA) at room temperature for 2 h. Following three washes of 5 min each in 1 × TBS containing 0.1% Tween 20, proteins were incubated with alkaline phosphatase (AP)-conjugated polyclonal goat anti-mouse IgG (Sigma-Aldrich Corp., St. Louis, MO, USA) and visualized using the NBT/BCIP^®^ solution (Sigma-Aldrich Corp., St. Louis, MO, USA).

### 2.4. Protein Purification

All GFP-fused proteins were purified by the GFP-TRAP-A purification kit (Chromo Tek, Rosemont, IL, USA) following the manufacturer’s instructions. Thereafter, the GFP-TRAP bound fraction was resolved by SDS-PAGE and stained with the EZBlue™ gel staining reagent (Sigma-Aldrich Corp., St. Louis, MO, USA).

### 2.5. Mass Spectrometric Analysis

Proteins of interest were excised from the stained SDS-PAGE gels, followed by in-gel digestion with trypsin, and analysis by nano-LC/MS/MS according to standard protocols. Samples were prepared as previously described by Granvogl et al. [24]. Mass spectrometry was carried out at the fingerprints proteomics facility of the University of Dundee and the Biomedical Sciences Research Complex mass spectrometry and proteomics facility of the University of St Andrews. 

### 2.6. High Throughput Sequencing 

The infiltration patches were harvested eight days after the infiltration of *N. benthamiana* with viruses. Total RNA was extracted from the collected samples with TRIzol reagent (Invitrogen, Waltham, MA, USA) in line with the manufacturer’s instructions. Sequencing libraries were prepared using NEBNext^®^ Ultra^TM^ RNA Library Prep Kit for Illumina^®^ (New England Biolabs, Ipswich, MA, USA) following the manufacturer’s recommendations. Meanwhile, index codes were added to attribute sequences to each sample. The library quality was assessed using the Agilent Bioanalyzer 2100 system. Deep sequencing was performed on the Illumina HiSeq 4000 platform (Illumina, San Diego, CA, USA) by Tianjin Novogene Bioinformatic Technology Co., Ltd. (Tianjin, China).

Raw reads of the viral vector from Hiseq 4000 sequencing were quality trimmed. The remaining reads were mapped to P3N-PIPO sequences of TuMV and WYMV using bowtie2 (version 2.4.1) with default parameters. Later, the mapped reads to identify insertion/deletion (indel) were performed by custom Perl scripts and Linux shell bash scripts.

## 3. Results

### 3.1. Frameshift Expression of P3N-PIPO in N. benthamiana by PVX-Based Vector

To test whether the PVX-based vectors were suitable for P3N-PIPO frameshift expression or not, we inserted the WYMV P3N-PIPO(WT) and TuMV P3N-PIPO(WT) into the PVX-based vector pgR106, which then named PVX-GFP (*Asc*I/*SnaB*I; *Sal*I) (Figure 1). It was expected that the frameshift expression of the constructs would result in the trans-frame fused expression of P3N-PIPO-GFP, in which GFP fluorescence was detected under a fluorescence microscope. 

**Figure 2:**
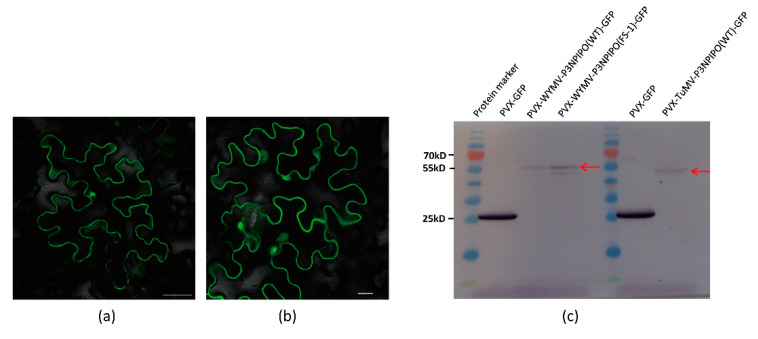
Frameshift expression of P3N-PIPO(WT) in *N. benthamiana* leaf cells. (**a**) PVX-TuMV-P3N-PIPO(WT)-GFP expressed in the epidermal cells treated eight days post-agroinfiltration. (**b**) PVX-WYMV-P3N-PIPO(WT)-GFP expressed in the epidermal cells treated eight days post-agroinfiltration. Bars, 50 μM. (**c**) Immunodetection of the frameshift expression of PVX-TuMV-P3N-PIPO(WT)-GFP and PVX-WYMV-P3N-PIPO(WT)-GFP in *N. benthamiana*.

As a result, these constructs were expressed in *N. benthamiana*, and the fluorescence of P3N-PIPO-GFP was successfully detected by Laser Scanning Confocal Microscopy (LSCM) (Figure 2a,b). 

To further confirm the expression of P3N-PIPO-GFP, we utilized a GFP antibody to detect the frameshift expression of P3N-PIPO. Additionally, an in-frame control, where the predicted shift site GGA_AAA_AAT_C was mutated to GGA_AAA_AAA_T to force the expression of WYMV P3N-PIPO(FS-1)-GFP, was prepared to indicate the approximate size (56 kD) at which the frameshift protein should theoretically migrate in the gels. Using PVX-GFP as a positive control, the frameshift expression of WYMV P3N-PIPO(WT)-GFP and TuMV P3N-PIPO(WT)-GFP was successfully detected (Figure 2c).

### 3.2. Mass Spectrometry Revealed That Frameshifting Used in P3N-PIPO(WT)-GFP Expression Took Place at the G_2_A_6_ Site

To determine the precise sites and direction of the frameshift expression of P3N-PIPO, total proteins were extracted from *N. benthamiana* infected with PVX-TuMV-P3N-PIPO(WT)-GFP and PVX-WYMV-P3N-PIPO(WT)-GFP. Later, the frameshift proteins were affinity-purified by GFP-TRAP beads and separated through SDS-PAGE. Afterwards, gel slices containing frameshift proteins were excised and digested with trypsin and were analyzed by mass spectrometry.

**Figure 3:**
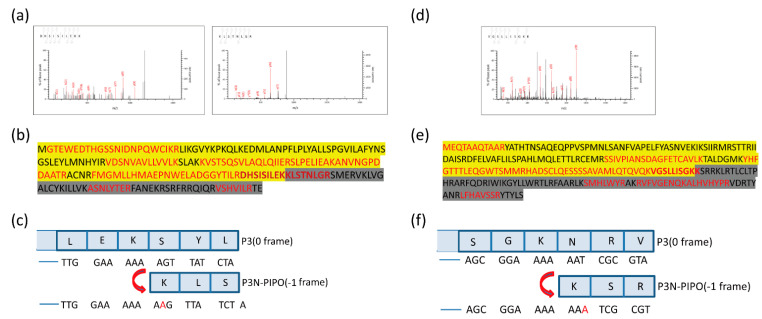
Mass spectrometry of the PVX-TuMV-P3N-PIPO(WT)-GFP and PVX-WYMV-P3N-PIPO(WT)-GFP frameshift expression proteins. (**a**) MS/MS fragmentation spectra of the shift site peptides DHSISILEKK and KLSTNLGR in TuMV-P3N-PIPO(WT). (**b**) The complete amino acid sequence of TuMV P3NPIPO. The peptides before the G_2_A_6_ site are highlighted in yellow. Amino acids encoded by the –1 frame are highlighted in dark. (**c**) Nucleotide sequence around the G_2_A_6_ shift site, and conceptual amino acids translated in the 0 and –1 reading frames in TuMV-P3N-PIPO. (**d**) MS/MS fragmentation spectrum of the shift site peptide VGSLLISGKK in WYMV-P3N-PIPO(WT). (**e**) The complete amino acid sequence of WYMV P3NPIPO. The peptides before the G_2_A_6_ site are highlighted in yellow. Amino acids encoded by the −1 frame are highlighted in dark. (**f**) Nucleotide sequence around the G_2_A_6_ shift site, and conceptual amino acids translated in the 0 and −1 reading frames in WYMV-P3N-PIPO.

Altogether, 10 unique peptides of TuMV P3N-PIPO and nine unique peptides of WYMV P3N-PIPO were identified, including peptides located in both the upstream and downstream predicted shift sites (Figure 3 and File S1). As the predicted shift site contains the trypsin digestion sites (arginine and lysine), it is difficult to obtain the tryptic peptide containing the predicted shift site. If there is an acidic amino like aspartic acid or glutamic acid in the direct neighborhood of the digestion site, the rate of hydrolysis is diminished [25]. Fortunately, glutamic acid was found near the digestion site at the predicted shift site of TuMV P3N-PIPO-GFP, suggesting the successful identification of two peptides spanning the shift site (Figure 3a). The peptides DHSISILEKK and KLSTNLGR showed that the frameshifting used in TuMV P3N-PIPO-GFP expression occurred at the G_2_A_6_ site in the –1 directions (Figure 3c). Another peptide of WYMV P3N-PIPO-GFP, VGSLLISGKK, contained the shift site, which showed that the frameshifting used in WYMV P3N-PIPO-GFP expression took place at the G_2_A_6_ site as well (Figure 3d,f). 

### 3.3. Deep Sequencing Confirmed the Adenosine Insertion at the G_2_A_6_ Site of P3N-PIPO

To further investigate the expression mechanism of P3N-PIPO at the transcriptional level, a transcriptomics analysis of PVX-P3N-PIPO-infected plants was performed by high-throughput sequencing.

**Figure 4:**
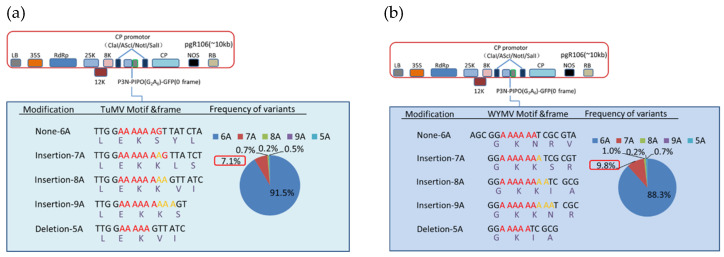
Indel frequencies derived from deep sequencing at the G_2_A_6_ site of P3N-PIPO of TuMV and WYMV fused to GFP and expressed from a PVX vector in *N. benthamiana*. The P3N-PIPO-GFP constructs of TuMV (**a**) and WYMV (**b**) are depicted schematically. Details of the nucleotide sequences and resulting amino acid sequences of the translation products, as well as the RNA slippage frequencies detected for each modification are shown. Color codes in the pie charts refer to insertions (7A, 8A, 9A) or deletion (5A) compared to the wild type sequence (6A) of P3N-PIPO. The slippage frequency associated with a single adenosine insertion (7A) is highlighted in the red rectangular boxes.

According to our results, various deletions and insertions of adenosine could be detected at the G_2_A_6_ site of P3N-PIPO of TuMV and WYMV. Besides, single adenosine insertion was the most abundant transcript editing form, and there was 7.1% and 9.8% of the transcript containing an adenosine insertion at the G_2_A_6_ site of TuMV and WYMV, respectively (Figure 4, Files S2 and S3). By contrast, the percentage of other transcript editing forms was below 1% (Figure 4). Combined with the mass spectrometry data, these results indicated that transcriptional slippage must be the expression mechanism of P3N-PIPO.

### 3.4. Hexa-Adenosine at G_2_A_6_ Site Was Necessary for the Frameshift Expression of WYMV P3N-PIPO 

**Figure 5:**
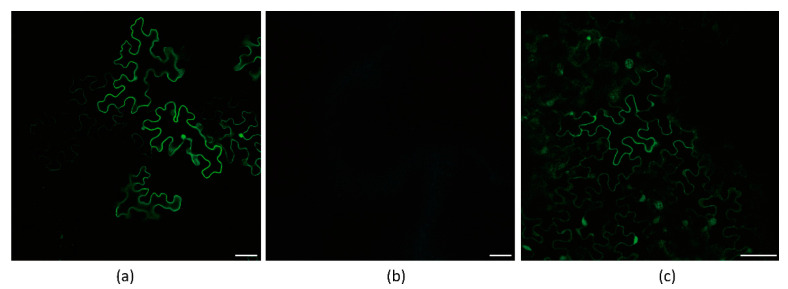
Frameshift expression of WYMV P3N-PIPO mutants in *N. benthamiana* leaf cells under confocal microscopy. (**a**) PVX-WYMV P3N-PIPO-WT-GFP expressed in the epidermal cells treated eight days post-agroinfiltration; (**b**) PVX-P3N-PIPO-M1-GFP expressed in the epidermal cells treated eight days post-agroinfiltration; (**c**) PVX-P3NPIPO-M2-GFP expressed in the epidermal cells treated eight days post-agroinfiltration. Bars, 50 μm.

To determine whether Hexa-adenosine was necessary for the frameshift expression of WYMV P3N-PIPO, two WYMV P3N-PIPO mutants were constructed, in which GGAAAAAA was mutated to GGACGCAA and CTAAAAAA, respectively. Thereafter, the frameshift expression of P3N-PIPO was monitored in inoculated *N. benthamiana* leaves under confocal microscopy. For wild-type P3N-PIPO (WT), GFP fluorescence was easily detected from 4 to 5 dpi in the infiltration area (Figure 5a). For P3N-PIPO M1, no GFP fluorescence was detected in the infiltration area at 5 and 10 dpi (Figure 5b), while for P3N-PIPO M2, GFP fluorescence was easily detected in the infiltration area at 5 dpi (Figure 5c). The behavior of P3N-PIPO M2 was similar to WT. These results indicated that Hexa-adenosine was important for the maintenance of P3N-PIPO frameshift expression. 

## 4. Discussion

The family *Potyviridae* comprises 10 genera and over 210 plant-infecting virus species, which can infect the most economically important crops in the world [26]. Most viruses in this family have single-stranded monopartite positive-sense RNA genomes, except for bipartite bymoviruses [2,26]. To date, studies on P3N-PIPO frameshift expression have been limited in the genus *Potyvirus*, and it remains unknown whether a similar frameshift expression of P3N-PIPO occurs in other genera in *Potyviridae*.

The in vivo frameshift expression of TuMV P3N-PIPO was proved by immunoblotting. Several reports have provided transcriptional evidence to support that P3N-PIPO is generated by polymerase slippage in a G_2_A_6_ conserved motif in potyviruses [2,19,27,28,29], and the single nucleotide insertion rate varies from 0.8 to 2.1% during the expression of P3N-PIPO [2,19]. Due to the extremely low expression level of P3N-PIPO in virus-infected plants, it is challenging to detect P3N-PIPO expression in vivo, let alone to identify the peptides spanning the slippery site.

Therefore, the PVX-based expression vector was used in this study to achieve an abundant P3N-PIPO expression for analysis, and TuMV P3N-PIPO(G_2_A_6_) was cloned into the PVX-based expression vector to test the validity of this approach. Our results indicated that the PVX-based expression vector was an excellent tool to study the expression mechanism of P3N-PIPO. In addition, peptides spanning the slippery site of P3N-PIPO were identified by mass spectrometry, which provided the first direct evidence for this frameshift expression. 

To investigate the differences in the expression mechanism of P3N-PIPO between different genera in the family *Potyviridae*, P3N-PIPO(G_2_A_6_) of a bymovirus (WYMV) was cloned into the PVX-based expression vector using the same approach. Integrated proteome and transcriptome analyses confirmed that both P3N-PIPOs of TuMV and WYMV were expressed in the G_2_A_6_ conserved motif by means of transcriptional slippage.

In this study, the PVX-based vector system was successfully developed and proved to be an efficient and convenient tool for exploring the P3N-PIPO slippage efficiency. Furthermore, we provided the first consolidated evidence to support that P3N-PIPO of viruses in the genus *Bymovirus* showed frameshift expression in the G_2_A_6_ conserved motif. All in all, the PVX-based system developed in this study contributed to future research on the functional study of P3N-PIPO in *Potyviridae*.

## Data Availability

The data presented in this study are available on request from the corresponding author.

## Supplementary Materials

The following are available online at https://www.mdpi.com/article/10.3390/v13071247/s1, File S1: The peptides information of TuMV-P3N-PIPO(WT)-GFP and WYMV-P3N-PIPO(WT)-GFP identified by mass spectrometry analysis; File S2: The counts of indel sequences of PVX-TuMV-P3N-PIPO(WT)-GFP; File S3: The counts of indel sequences of PVX-WYMV-P3N-PIPO(WT)-GFP; Table S1: Primers used in this study.
