# Supplementary material for: Integrated Proteomics and Transcriptomics Analyses Reveal the Transcriptional Slippage of a Bymovirus P3N-PIPO Gene Expressed from a PVX Vector in Nicotiana benthamiana
Source: Viruses. 2021 Jun 26;13(7):1247. doi: 10.3390/v13071247 (PMC8310318; doi:10.3390/v13071247)
Supplement: Supplementary file 1 [file viruses-13-01247-s001.zip › viruses-1266168-SI/supplementary files/Table S1.pdf]

Table S1 Primers used in this study

Serial Number	Name of Primers	Sequences
**1**	GFP-F(*Asc*I/*SnaB*I)	5'-AGGCGCGCCTACGTAGTGAGCAAGGGCGAGGAGC-3
**2**	GFP-R(*Sal*I)	5'-GCGTCGACTTACTTGTACAGCTCGTCCATGCC-3'
**3**	WYMV-P3N-PIPO-F	5'-CCATCGATATGGAGCAGACAGCAGCCCAG-3'
**4**	WYMV-P3N-PIPO-R	5'-CTACGTACTACGAAAGATAGGTATAGCGCGAA-3'
**5**	TuMV-P3N-PIPO-F	5'-CCATCGATATGGGAACAGAATGGGAGGACAC-3'
**6**	TuMV-P3N-PIPO-R	5'-CTACGTACTCCGTTCGTAAGATGACATGACTG-3'
**7**	WYMV-P3N-PIPO-FS-1-F	5'-CTCCCTCTTAATCAGCGGAAAAAAATCGCGTAGAAAGT-3'
**8**	WYMV-P3N-PIPO-FS-1-R	5'-ACTTTCTACGCGATTTTTTTCCGCTGATTAAGAGGGAG-3'
**9**	WYMV-P3N-PIPO-M1-F	5'-CTCCCTCTTAATCAGCGGACGCAATCGCGTAGAAAGT-3'
**10**	WYMV-P3N-PIPO-M1-R	5'-ACTTTCTACGCGATTGCGTCCGCTGATTAAGAGGGAG-3'
**11**	WYMV-P3N-PIPO-M2-F	5'-CTCCCTCTTAATCAGCCTAAAAAATCGCGTAGAAAGT-3'
**12**	WYMV-P3N-PIPO-M2-R	5'-ACTTTCTACGCGATTTTTTAGGCTGATTAAGAGGGAG-3'
